# Emotion-Based Cognition in Mice Is Differentially Influenced by Dose and Chemical Form of Dietary Docosahexaenoic Acid

**DOI:** 10.3390/nu9090993

**Published:** 2017-09-08

**Authors:** Kevin D. Laugero, Yuriko Adkins, Bruce E. Mackey, Darshan S. Kelley

**Affiliations:** 1 US Department of Agriculture, Agricultural Research Service, Western Human Nutrition Research Center, Davis, CA 95616, USA; kevin.laugero@ars.usda.gov (K.D.L.); yuriko.adkins@ars.usda.gov (Y.A.); 2 US Department of Agriculture, Agricultural Research Service, Western Regional Research Center, Albany, CA 94710, USA; bruce.mackey@ars.usda.gov

**Keywords:** phospholipid docosahexaenoic acid, triglyceride docosahexaenoic acid, *n*-3 polyunsaturated fatty acids, diet, cognitive function, behavior, mice

## Abstract

Docosahexaenoic acid (DHA) is a major constituent, and primary omega-3 fatty acid, in the brain. Evidence suggests that DHA consumption may promote cognitive functioning and prevent cognitive decline, and these effects may be particularly relevant in the context of fear or stress. However, the potency and efficacy of dietary DHA may depend on the form of DHA (e.g., phospholipid; PL vs. triglyceride; TG). In this study, we compared in mice the effects of consuming PL and TG forms of DHA on associative, avoidance (fear) based learning and memory. Diets consisted of either no DHA or 1%, 2%, and 4% PL- or TG-DHA. After 4 weeks on the test diets (*n* = 12/group), we used the 3-day passive avoidance (PA) and elevated plus maze (EPM) to examine fear and fear-associated learning and memory. We found a significant (*p* < 0.05) diet by time interaction in the PA and EPM. Compared to the control and the 1% TG-DHA group, mice consuming the diet supplemented with 1% PL-DHA displayed a significantly greater latency by test day 2 in the 3-day PA. No differences in latency between any of the groups were observed during trials 1 and 3. Mice consuming the 2% PL-DHA diet spent significantly more time frequenting the open arms during the first minute, but not the last 4 min, of the test. Compared to all other groups, mice fed the 4% TG-DHA diet had increased spleen, liver, and visceral fat weight. Consumption of the lower dose PL-DHA may confer enhanced efficacy, particularly on fear-based learning behavior.

## 1. Introduction

Docosahexaenoic acid (DHA; 22:6*n*3) is the primary omega-3 (*n*-3) fatty acid, in the brain. This long chain polyunsaturated fatty acid (PUFA) makes up a high percentage of the neuronal membrane phospholipids and, in turn, affects neuronal signaling, neurogenesis, synaptic activity, and overall neurological function [1,2]. DHA also has protective effects against neuroinflammation [3,4]. Inflammation and disrupted neuronal function related to membrane composition and structure are possible mediators of psychiatric and neurodegenerative diseases [2,5,6]. Thus, it is not surprising that decreases in brain DHA content are linked to suboptimal functioning of the brain and a number of central nervous system (CNS) diseases, including Alzheimer’s disease (AD), Parkinson’s disease (PD), and depression [7,8,9,10].

Due to an aging population and the modern impact of Alzheimer’s disease and other cognitive-related conditions, there have been major scientific efforts to better understand the biological relationships between DHA status and cognitive function. Evidence suggests that declining DHA levels in the brain, which occurs with aging [11,12] and Alzheimer’s disease [13,14], increase the risk of cognitive decline and certain psychiatric diseases, such as major depression [2,15,16,17]. In fact, due to the limited efficacy and side effects of medications for these conditions, improved and alternative treatment options are needed. Dietary DHA has been considered as one type of alternative or complimentary treatment option [2,15,16,17]. In general, evidence suggests that diet affects neuronal structure and function over the life course [1,7,18]. Diet is thought to influence cognitive function in part by affecting synaptic plasticity, neural connections, and neurogenesis [19]. Inflammation, also influenced by nutrition, may contribute to detriments in cognitive function and related diseases. The ratio between the *n*-6 PUFA, such as arachidonic acid (AA; 20:4*n*6) and *n*-3 PUFA (DHA) is one of the major factors that determine brain inflammation and membrane function [20,21]. A decrease in plasma concentration of *n*-3 PUFA and an increase in the ratio between *n*-6 and *n*-3 PUFA may increase the risk of brain disorders [21]. Other epidemiological studies reported decreased risk of brain inflammatory diseases with increased intake of fish [22]. Recent intervention studies have demonstrated that DHA supplementation delayed the development of some of the inflammatory diseases of the CNS [22,23,24]. DHA consumption has been shown to facilitate memory [25] and cognitive function throughout life [2]. DHA is also suspected of having beneficial effects on affective processes and mood [26].

However, while evidence supports DHA consumption as a preventative factor limiting cognitive impairment, the results remain inconclusive [2,23,27] and more work to understand factors such as the dose given, exposure time required, and the source and form of DHA, is needed to optimize the potential health benefits of consuming DHA and other PUFAs. Even though epidemiological and animal studies support a role for dietary DHA in the prevention and treatment of cognitive dysfunction and disease, controlled clinical feeding studies of DHA effects on declining cognition in the elderly have not been promising [28,29,30]. While several reasons may explain these discrepancies (e.g., dose, time and route of exposure, test performed, health status), the origin and form of dietary DHA may play a key role. The accretion of DHA from the phospholipid (PL) form has been demonstrated to be greater than that of the triglyceride (TG) form in the brain, liver and kidneys [31,32]. It is possible that accretion of DHA is higher in the brain because of a specific transporter for the lysophosphatidyl choline-DHA (LPC-DHA) in the endothelial cells of the blood brain barrier [33]. An increase in tissue concentration of DHA from the PL form over that of the TG form suggests that the PL form should be more effective in improving DHA-mediated brain functions. It is not known if the PL form of DHA will be more or less potent than the TG form in facilitating cognitive function. In this study, we compared the effects of consuming PL- and TG-based DHA on associative avoidance (fear) based learning and memory in adult mice. From this study, we conclude that consuming comparatively smaller amounts of PL-DHA, but not TG-DHA, may confer greater efficacy, particularly on fear-associated learning behavior, and these effects may come with less negative side effects.

## 2. Materials and Methods

### 2.1. Experimental Design

The animal protocol was approved by the Institutional Committee for the Animal Use and Care of University of California, Davis (Animal Protocol #18911). All procedures were in compliance with the guidelines of the National Institutes of Health for experimental animals. Ten to 12-week-old, pathogen-free C57BL/6J female mice (Jackson Laboratory, Sacramento, CA, USA) were housed in a 2 mice cage separated by a barrier (each mouse had access to its own living quarters, food, and water) at the animal facility in the Mouse Biology Program (MBP) at UCD. Animals were acclimated to the laboratory chow diet for 7 days before they were randomly assigned to 1 of 7 experimental diets for the following 38 days (12 animals/group). A modified AIN93G diet was used as the control diet where corn oil replaced soybean oil; soybean oil contains ~7% alpha-linolenic acid (ALA; 18:3*n*3) versus corn oil contains ~1% ALA. This substitution controls for the elongation of dietary ALA to DHA that could potentially contribute to the DHA pool. The remaining 6 diets had a portion of the corn oil replaced with equivalent amounts of highly enriched PL- or TG-DHA (both >90% purity; Larodan Fine Chemicals, Malmo, Sweden). Concentrations of DHA (*w*/*w*) added to the diets were 1%, 2%, and 4% PL-DHA and 1%, 2%, and 4% TG-DHA. Total fat content in all diets was 8%. To prevent DHA oxidation, 0.002% (*w*/*w*) tert-butylhydroquinone was added to all diets and the pelleted diets were vacuum packaged in 125 g pouches (Envigo, Madison, WI, USA). All packaged diets were stored at −20 °C until use.

Trained technicians at MBP conducted the day-to-day animal care and tissue necropsies. Fresh pelleted diet (~5 g/mouse) was served daily and the food left from the previous day was weighed and recorded. Body weight was recorded every 7 days. Animals were terminated by CO_2_ asphyxiation after withholding food for 10 to 12 h. Tissues including the brain were collected, weighed, frozen in liquid nitrogen, and stored at −80 °C for future analysis.

### 2.2. Behavioral Tasks

After 28 days of feeding the experimental diets, the trained technicians at MBP performed the passive avoidance (PA) and elevated plus maze (EPM) tests. All behavioral testing was performed in a room separate from the room where mice were housed. The PA task is a fear-based test used to assess learning and memory. This task was conducted using a 2-chamber box (Med Associates, Saint Albans, VT, USA), which was equally partitioned by a white sliding door. The entire apparatus is 42 × 16 × 21 cm. One side of the box is lit by an 800 Lux bulb, and the mice can see through the chamber walls. The chamber is kept dark, with all walls covered. On each of the 3 test days, mice were individually placed into the lighted chamber. After 10 s, the partition was opened. When mice entered the dark chamber, the dividing door was closed and a 0.5 mA foot shock was delivered for 2 s via a grid floor. Eight seconds later, the mouse was placed back in its home cage. The time taken to enter the dark side of the chamber (latency) was recorded, and a maximum latency of 300 s was allowed. Twenty-four and 48 h later, the mice were tested again in the same fashion, but no shock was delivered on the 3rd trial (day 3). Trials 2 and 3 were used to examine learning and memory. Vocalizations were observed as a verification of the foot shock delivery. Between each test, feces and urine were removed from the test chambers. The chamber walls, floor, and shock grid were then thoroughly cleaned with 10% Nolvasan. The partition door was removed and the dark chamber cover was replaced.

The EPM test is based on the mouse’s natural aversion or fear of open spaces. Relative to the closed arms, total less time spent in or frequenting the open arms has long been used as a marker of enhanced anxiety. Furthermore, temporal analysis of the rodent’s exploratory behavior with respect to open and closed arms of the maze was used as an index of learning and memory [34]. In this study, 2 arms of the maze were closed and dark tinted while the other 2 arms were open. The maze was elevated to approximately 1 m above the floor of the testing room. Testing was performed with white lights off and under red lighting.

A plus maze Smart video tracking system (Panlab) was used to quantify time spent and entries into arms and center of the maze. Mice were placed onto the center platform of the maze and behavior was recorded for 5 min. Total amount of time spent in and entries into the arms and center of the maze were examined over the 5-min period. Additionally, over this same 5-min test, time spent in and entries into the arms and center were analyzed over 5, 1-min intervals. Arm entry was counted when all 4 paws entered an arm. Measurements assessed for each of the distinct zones (each arm and center platform) included: distance in zone (%); distance in zone (s); total distance; entries in zone; latency 1st entry into zone (s); resting time in zone (%); resting time in zone (s); time in zone (%); time in zone (s); fast time in zone (s); fast time in zone (%); slow time in zone (s); slow time in zone (%); max speed in zone; min speed in zone; mean speed in zone; and mean speed without resting in zone. After each test, the maze was cleaned using 70% ethanol. The total distance traveled was included in the statistical model to help control for effects of general locomotion.

### 2.3. Lipid Extraction and Quantification

Approximately 30 mg of powderized frozen brain tissue and ~60 mg of powderized diet were spiked with 5 μL butylated hydroxyl toluene/EDTA (0.2 mg/mL in 1:1 in methanol:water) and 20 μL of methanol containing a deuterated TG, a deuterated phosphatidylcholine, and a rare cholesteryl ester and fatty acid as extraction surrogates, and three 3 mm stainless steel balls (Retsch, Newtown, PA, USA). Lipids were extracted following a modification of the published protocols of Smedes [35] which uses stepwise cyclohexane/2-propanol/0.1 M ammonium acetate (10:8:11, *v*:*v*) extraction solvents. After the addition of the solvents, the Geno/Grinder (SPEX SamplePrep, Metuchen, NJ, USA) was used at 1200 rpm for a total of 3 min to disrupt the tissue and extract the lipids. Phase separation was achieved by adding 0.1 M ammonium acetate, vortexing, centrifuging for 5 min at 10,000× *g* at room temperature, and removing the top organic phase. The samples were extracted a second time and the solvent of the combined top organic phase was evaporated under vacuum. Total lipids were reconstituted in 1 mL of methanol and toluene (1:1). Aliquots of the total lipid extract (25 μL) were subjected to methanolic acid/base transesterification and the resultant fatty acid methyl esters were quantified by GC-MS as previously described [36].

### 2.4. Statistical Analysis

Mixed model analysis (SAS) in a repeated measures design was used to examine behavior in the PA test. For analysis of the EPM, total distance traveled was included in the statistical model as an independent variable in order to control for general locomotor effects. For temporal analysis of behavior in the EPM, a repeated measures design was used to test for group differences across 1 min epochs over the 5 min testing duration. For both behavioral tests, if we observed a significant diet by time interaction, we included a slice statement to examine effects of diet at each of the observation times. Analysis of covariance (ANCOVA) was applied to assess effects of diet on body weight, food intake, and excised tissues. For fatty acid analysis, log transformations were used for C16:0, C18:0, C18:2*n*6, C22:5*n*3 and C22:6*n*3. Ranks were used for C20:1*n*9, C20:5*n*3 and C24:1*n*9. The remaining variables were untransformed. Differences in body weight can confound interpretation of treatment differences in end tissue weight and mask specific effects on tissue weight that are independent of general, more global effects throughout the body. As is standard practice in animal studies [37,38], in order to assess whether differences in tissue weights between groups were not attributed to differences in body weight, we included final body weight as a covariate in the statistical analysis (ANCOVA). Therefore, the ANCOVA provides a way to examine body weight independent effects of the treatments on tissue weights. Tissue weight values are reported as least squared means, which reflect the mean tissue weights after removing effects due to body weight.

## 3. Results

### 3.1. Body and Tissue Weight and Food Intake 

**Figure 1:**
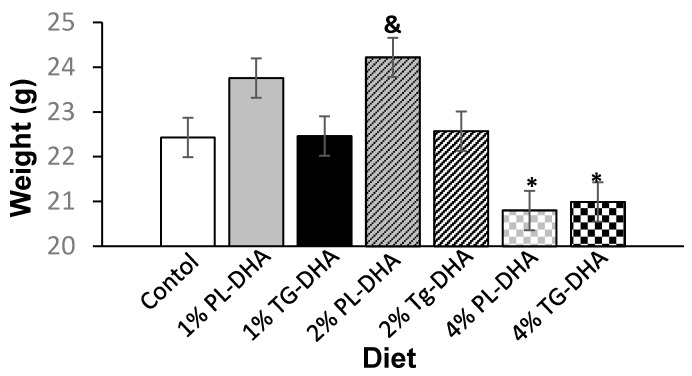
Final body weight, prior to the overnight fast. * Significantly (*p* < 0.05) different from 1% and 2% PL-DHA after Tukey multiple comparison tests. ^&^ Tended to be statistically (*p* = 0.08) different from control diet after Tukey multiple comparison tests.

**Figure 2:**
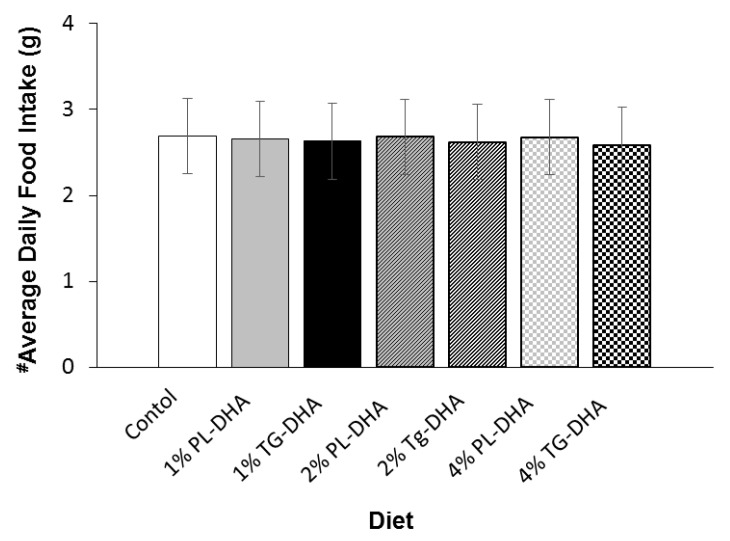
Average daily food intake did not differ between the diet groups. ^#^ adjusted for average daily body weight.

Initial body weights did not differ between the groups. The final, pre-fasted, body weight for 2% PL-DHA tended to be (*p* = 0.08) heavier than the final body weight of the control group (Figure 1). Mice fed the 4% PL-DHA and TG-DHA diets had lower (*p* < 0.05) final body weights compared to body weights of mice consuming the 1% and 2% PL-DHA diets. Tissue weights were statistically adjusted for the body weight taken immediately prior to euthanization and tissue harvest (Table 1). As noted in [37,38] and stated above, treatment effects on body weight can confound interpretation of treatment differences in tissue weight and mask specific effects on tissue weight that are independent of general, more global effects throughout the body. Therefore, in order to assess body weight independent treatment effects on tissue weights, we included body weight as a covariate to the statistical model to statistically remove treatment effects due to body weight. Body weight adjusted brain weights did not differ between the 2 forms of DHA for all 3 concentrations used; nor did they differ from the control group. There was a highly significant (*p* < 0.0001) effect of diet on body weight independent spleen weight. Body weight adjusted spleens weighed significantly (*p* < 0.03) more in both of the 4% DHA groups compared to all other groups. Furthermore, body weight adjusted spleens from the 4% TG-DHA group were significantly (*p* < 0.0015) heavier than spleens from the 4% PL-DHA group. After adjusting for differences in body weight, the 4% TG-DHA group had statistically (*p* < 0.05) heavier livers when compared to the livers of 1% PL- and TG-DHA groups. Livers from the 4% TG-DHA group tended to be heavier when compared to the livers of the 2% PL DHA (*p* = 0.0571) and 2% TG DHA (*p* = 0.0886) groups. Body weight adjusted mesenteric fat pad weights, but not subcutaneous or peri-uterine fat pad weights, were significantly (*p* = 0.0077) affected by diet. The 4% TG DHA group had significantly (*p* < 0.05) elevated mesenteric fat pad weight compared to the same fat pad in 1% and 2% PL-DHA groups. Body weight independent quadriceps and gastrocnemius, but not soleus, muscles were significantly (*p* < 0.05) affected by diet. Body weight adjusted quadriceps weighed significantly (*p* = 0.02) less in the 4% PL-DHA compared to the control group. The gastrocnemius muscle of mice fed the 4% PL-DHA weighed significantly (*p* < 0.05) less compared to those fed the control diet. No statistical differences between treatment groups in heart or eye weights were observed. No differences in average daily food intake were observed (Figure 2).

### 3.2. Behavioral Tests

**Figure 3:**
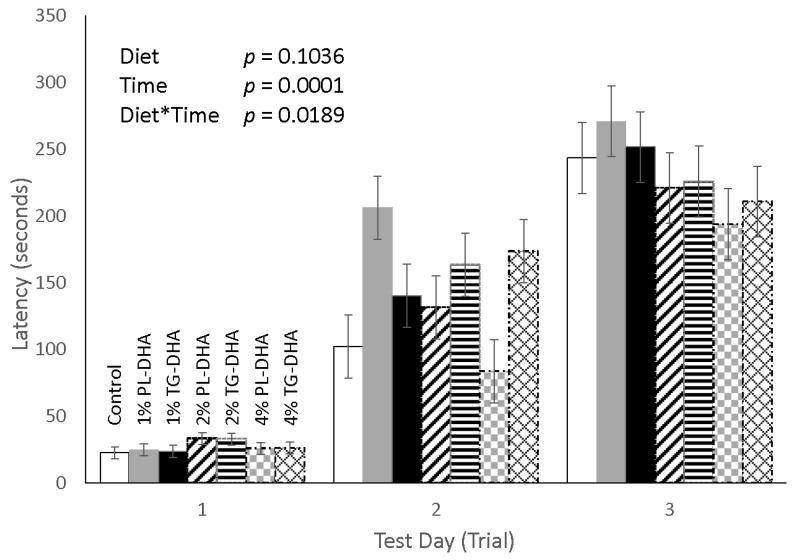
Passive avoidance test. A statistically significant (*p* = 0.0189) diet by time interaction was observed. Upon further inspection at each trial (test day) and compared to mice fed the control diet, mice fed a 1% phospholipid (PL) or 4% triglyceride (TG) derived DHA diet displayed significantly (*p* < 0.05) increased latency on trial 2 of the behavioral task. No latency differences between groups were found on trials 1 and 3.

**Figure 4:**
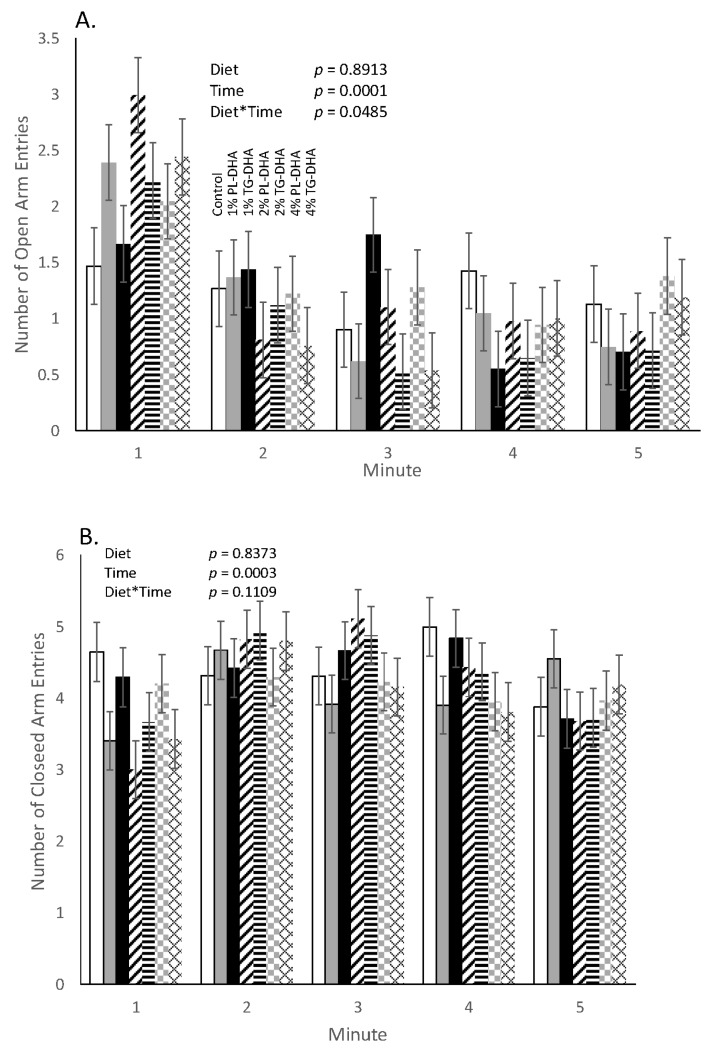
Elevated plus maze ((**A**) Open arm entries; (**B**) Closed arm entries). A statistically significant (*p* = 0.0485) diet by time interaction was observed for open arm entries. Upon further inspection at each minute and compared to controls, mice fed 2% phospholipid (PL)- or 4% triglyceride (TG)-DHA showed significantly (*p* < 0.04) more open arm entries during the first one minute epoch. Mice fed the 1% PL-DHA tended (*p* = 0.0513) to follow this same behavior pattern. No statistical group differences were observed for entries into the closed arms.

Overall, we found a significant (*p* = 0.0189) diet × time interaction for the passive avoidance test. Compared to the control and the 1% TG-DHA group, mice consuming the diet supplemented with 1% PL-DHA displayed a significantly greater latency by trial 2 (test day 2) in the 3-day passive avoidance test (Figure 3). Mice fed the 4% TG-DHA also showed greater latency in trial 2 when compared to the controls. No differences in latency between any of the groups were observed during trials 1 and 3. When examining the sum scores across the entire 5-min duration of the elevated plus maze, no differences were observed with regard to any of the maze parameters. However, when examining across one minute epochs over the 5-min elevated plus maze test, we found a significant (*p* = 0.0485) diet × time interaction for time spent in and number of entries into the open arms of the maze (Figure 4). Compared to the controls, mice consuming the 2% PL- (*p* = 0.0015) and 4% TG- (*p* = 0.0398) DHA diets entered the open arms more during the first minute, but not in the remaining 4 min, of the test. Mice fed the 1% PL-DHA tended (*p* = 0.0513) to follow this same behavior pattern. However, as noted above, mice fed the 4% TG-DHA diet also displayed body weight loss and elevated spleen weights. Across groups, this pattern of entry into the open arms was inversely reflected by the entry pattern in the closed arms of the maze.

### 3.3. Brain Fatty Acid Composition

Brain *n*-3 and *n*-6 PUFA concentrations significantly differed (*p* < 0.05) between mice fed the control diet and mice fed the DHA enriched diets (Table 2). Compared to brains of mice fed the control diet, brain lipid concentrations of total *n*-6 PUFA, 20:4*n*6 (AA), and 22:4*n*6 fatty acids were significantly (*p* < 0.05) lower in mice fed the 1%, 2%, and 4% DHA enriched diets. Furthermore, the brain lipid 18:2*n*6 (linoleic acid, LA) concentrations were lower in mice fed 4% PL-DHA compared to 4% TG-DHA enriched diets. While brain concentrations of 22:6*n*3 (DHA) did not differ between any of the groups, consuming diets enriched in 1%, 2%, and 4% DHA significantly (*p* < 0.05) and markedly elevated brain concentrations of 20:5*n*3 and 22:5*n*3 fatty acids. Relative to mice fed the control diet, mice fed the 4% TG-DHA enriched diets had significantly higher total *n*-3 PUFA brain concentrations. The ratio of *n*-6:*n*-3 fatty acid concentrations in brain was significantly lower in mice fed the 1%, 2%, and 4% DHA diets, independent of whether the DHA was in the PL or TG form.

## 4. Discussion

DHA is the most abundant *n*-3 PUFA in the brain, and humans rely on dietary sources of this PUFA to support healthy neuronal structure and function. Deficiencies in *n*-3 PUFA, including DHA, have been linked to impaired learning and memory, as well as other mental health problems [1]. Some evidence suggests that dietary DHA supplementation may promote cognitive function and prevent cognitive decline [2,25]. However, while evidence supports DHA consumption as a preventative factor limiting cognitive impairment or promoting learning and memory, the results remain inconclusive [2,23,27]. Given that accretion of DHA in the PL form was shown to be greater than that of the TG form in the brain, liver and kidneys [31,32], the origin and form of DHA may determine its potency and efficacy, with the PL form potentially having enhanced effects. In this study, we compared the effects of consuming PL- and TG- DHA on behavioral assays of fear (EPM) and associative avoidance (fear) based learning and memory (PA) in healthy adult mice.

Our PA results suggest that mice fed diets enriched with the lowest test dose (1%) of PL-DHA more rapidly learned to avoid an unpleasant stimulus (shock), even though this avoidance behavior conflicts with moving into a location of the testing apparatus that is typically preferred and considered safer by rodents. This apparent effect to expedite associative learning was not observed in mice consuming diets with 1% or 2% TG-DHA. While the 4% TG-DHA group displayed faster learning in the PA test, this group also had elevated mesenteric fat, spleen, and liver weights. Elevated visceral fat, spleen, and liver weight has been linked to chronic inflammation and poor health [39,40,41]. Therefore, these findings suggest that increasingly higher doses of DHA may actually limit its utility in promoting brain functions related to learning and memory. As an alternative interpretation, increased latency in the PA could be indicative of enhanced fearfulness over time. However, there is some evidence that DHA alters stress pathways and dampens anxiety (see discussion, below), which can inhibit cognitive performance.

Similar to our findings in the PA, a lower (2%) dose of the PL-DHA and highest dose (4%) of the TG-DHA had effects on behavior in the EPM. Mice fed these diets displayed a higher frequency of open arm entries. However, this occurred in the first minute of the 5-min trial and declined to the open arm entry levels observed in all other groups by the second minute. On the one hand, increased open arm entry during the first minute may suggest that 2% PL-DHA and 4% TG-DHA consumption had reduced anxiety levels in those mice, since more time spent in or frequenting the open arms has typically been interpreted as indicating less anxiety or anxiolytic effects. This interpretation is consistent with previous reports showing that dietary DHA can blunt stress reactions [42,43,44] and stress or fear associated cognitive performance [45,46,47]. In fact, it has been suggested that tasks involving elevated stress or fear may more effectively distinguish cognitive effects of DHA [44]. Moreover, it was shown that DHA and *n*-3 rich diets affect neurophysiological pathways, such as the hypothalamic–pituitary–adrenal axis and hippocampus, that regulate behavioral and emotional reactions to stress [42,46,48,49,50]. Therefore, these prior reports along with our findings suggest that DHA consumption in this study may have reduced general anxiety and elevated cognitive performance.

However, as noted, the effects of DHA on open arm entry were confined to the first minute of the 5-min test. These observations might suggest that mice fed DHA were more inclined to initially explore a novel, including potentially dangerous, condition, as a way to escape the maze generally [34]. As such, they might be more equipped to learn about the spatial configuration of the maze and, in turn, where to best situate them later. Yet, another possible interpretation of these findings is that these mice were more anxious going into the task. Studies have shown that stress-reactive mice characterized by high anxiety (e.g., BALB/c) display increased entries into the open arms [34]. This counterintuitive behavioral profile has been hypothesized to represent the animal’s early attempt to generally escape the whole maze. However, it should be noted that those mice have also been reported to be more active, in general, in this test [51]. In our examination, we statistically controlled for total distance traveled. Given prior research supporting anti-stress and anti-depressant [15,16,52] effects of dietary *n*-3, we believe it is more likely that our findings in the EPM reflect reduced fear or emotional reactivity. Since fear or anxiety can impede cognitive performance, our findings in the EPM may partly explain the positive impact of DHA on cognitive performance in the PA. Further testing is needed to better understand if there is a common pathway linking the apparent cognitive and anti-stress effects of DHA.

In general, our results in the PA and EPM corroborate previous reports showing that *n*-3 consumption improves associative learning and memory, including fear-based learning, in rodents [44,53]. While these studies used a variety of types and sources of *n*-3, predominantly in the TG, mixed PUFA, or ethyl ester form, our results suggest that, compared to TG-DHA, consuming PL-DHA may more potently buffer against fear and/or enhance associative (fear) based learning. There is some research reporting on the effects of PL derived *n*-3. For example, DHA-rich PL prepared from pig brain were shown to enhance cognition and dampen anxiety in aged mice [54]. In the present study, while the highest dose of TG-DHA appeared to have similar effects to those of lower doses of PL-DHA in the PA and EPM tasks, consumption of the diet containing the highest dose of TG-DHA also appeared to come at a cost, as reflected by elevated visceral fat, spleen, and liver weight. Therefore, our findings highlight a need to further examine the importance of form and dose of DHA when examining its potential neurological benefits.

While we observed some differences in the effects of PL-DHA and TG-DHA on behavioral responses and tissue weights, neither of the two forms of DHA used increased its concentration in the brain when compared with that in the control group. Furthermore, there was no effect of the dose of DHA used on its accretion in the brain. The lack of increase in brain DHA by either form of dietary DHA most likely was due to the use in our study of adult mice, which may have brain cells already saturated with DHA. Saturation of brain cells with DHA may also be responsible for the lack of a dietary dose dependent increase in brain DHA. It is possible that some of the dietary effects observed in our study resulted from phosphatidyl choline or changes in the brain concentrations of other fatty acids. The lack of difference in the brain accretion of the PL-DHA and TG-DHA in our study varies from that reported in two studies with rats [31,32]. While these differences in our results may be due to different species, other factors may also have contributed. In those rat studies the DHA was gavaged instead of being fed in the diet and the dose of DHA used was 2 mg/Kg BW ([31]) and 8 mg/Kg BW ([32]) while, in our study, the lowest dose was approximately 150 mg/Kg BW. Further studies using lower doses of DHA and mice deficient in DHA are needed to determine if there is a difference in the accretion of the two forms of DHA used in our study.

In summary, a unique feature of our study is that we compared the effects of differing doses of PL- and TG-DHA on cognition and anxiety, as well as on body weight and relative tissue weight, which is a commonly used and sensitive marker of metabolic response or toxicity [37,38]. Other strengths include the use of highly purified PL- and TG-DHA, adequate power, and controlled feeding and experimental execution by trained technicians. Our study also had its limitations; we examined the lipid composition of the brain as a whole and not in specific areas. There is evidence for regional distribution of DHA and other fatty acids in the brain, and this can be influenced by diet [55]. Therefore, we suspect that regional differences in brain fatty acids, in response to dietary PL- and TG-DHA may explain behavioral differences found in this study. It is, of course, also possible that some alternative, indirect, pathway linking body and brain (e.g., liver, gustatory), the relatively short duration of the study, and the age of the mice mediated the behavioral effects observed in this study. Additional mechanistic studies are warranted.

## 5. Conclusions

We found dose-dependent effects of DHA consumption on behavior and metabolism. DHA consumption positively influenced associative, fear-based learning, but consuming higher doses of DHA appear to induce a metabolic profile consistent with inflammation; reduced body weight and elevated body weight independent spleen and liver weight. Consuming comparatively smaller amounts of PL-DHA, but not TG-DHA, may confer greater efficacy, particularly on learning behavior, while any positive effects of larger doses may come at a metabolic cost.

## Figures and Tables

**Table 1:** Tissue weights of mice fed experimental diets.

Tissue (g)	Control	1% PL-DHA	1% TG-DHA	2% PL-DHA	2% TG-DHA	4% PL-DHA	4% TG-DHA
#Mesenteric Fat	0.205 ± 0.017 ^ab^	0.174 ± 0.017 ^a^	0.203 ± 0.017 ^ab^	0.189 ± 0.018 ^a^	0.217 ± 0.017 ^ab^	0.225 ± 0.017 ^ab^	0.244 ± 0.017 ^b^
Subcutaneous Fat	0.654 ± 0.084	0.564 ± 0.085	0.751 ± 0.084	0.539 ± 0.086	0.655 ± 0.084	0.626 ± 0.085	0.689 ± 0.085
Peri-Uterine Fat	0.755 ± 0.075	0.659 ± 0.076	0.790 ± 0.075	0.643 ± 0.077	0.770 ± 0.075	0.767 ± 0.076	0.824 ± 0.076
Brain	0.463 ± 0.007	0.461 ± 0.007	0.471 ± 0.007	0.450 ± 0.007	0.467 ± 0.007	0.457 ± 0.007	0.462 ± 0.007
#Liver	0.787 ± 0.042 ^ab^	0.707 ± 0.043 ^a^	0.705 ± 0.042 ^a^	0.744 ± 0.043 ^ab^	0.751 ± 0.042 ^ab^	0.789 ± 0.043 ^ab^	0.849 ± 0.043 ^b^
#Spleen	0.053 ± 0.003 ^a^	0.058 ± 0.003 ^a^	0.056 ± 0.003 ^a^	0.061 ± 0.003 ^a^	0.061 ± 0.003 ^a^	0.074 ± 0.003 ^b^	0.090 ± 0.003 ^c^
#Quadriceps	0.151 ± 0.006 ^a^	0.145 ± 0.007 ^ab^	0.142 ± 0.007 ^ab^	0.137 ± 0.007 ^ab^	0.146 ± 0.007 ^ab^	0.126 ± 0.007 ^b^	0.143 ± 0.016 ^ab^
#Gastrocnemius	0.115 ± 0.003 ^a^	0.113 ± 0.003 ^ab^	0.120 ± 0.003 ^ab^	0.114 ± 0.003 ^ab^	0.119 ± 0.003 ^ab^	0.104 ± 0.003 ^b^	0.113 ± 0.003 ^ab^
Soleus	0.006 ± 0.001	0.007 ± 0.001	0.007 ± 0.001	0.006 ± 0.001	0.007 ± 0.001	0.008 ± 0.001	0.007 ± 0.001
Heart	0.098 ± 0.008	0.096 ± 0.008	0.092 ± 0.007	0.099 ± 0.009	0.097 ± 0.001	0.910 ± 0.001	0.089 ± 0.008
Eyes	0.039 ± 0.001	0.040 ± 0.002	0.040 ± 0.002	0.041 ± 0.001	0.041 ± 0.002	0.041 ± 0.003	0.041 ± 0.003

Data are least squared mean ± standard error, *n* = 12. Means without common letters within the same row differ significantly. Tissue weights were adjusted for final body weight in the statistical model. As noted in [37,38], treatment effects on body weight can confound interpretation of treatment differences in tissue weight and whether these effects on tissue weight are independent from general, more global effects throughout the body. Therefore, in order to assess whether the differences in tissue weights between groups were not attributed to differences in body weight, final body weight was a covariate in the statistical analysis (analysis of covariance; ANCOVA). **^#^** Statistical Effect of Diet (*p* < 0.05).

**Table 2:** Fatty acid composition of brain lipids in mice fed experimental diets.

Fatty Acid μmol/g	Control	1% PL-DHA	1% TG-DHA	2% PL-DHA	2% TG-DHA	4% PL-DHA	4% TG-DHA
SFA							
16:0	26.44 ± 1.22	24.24 ± 1.00	26.70 ± 1.31	26.24 ± 1.13	26.37 ± 1.12	25.25 ± 0.99	27.37 ± 1.68
18:0	23.29 ± 0.84	20.88 ± 0.89	22.69 ± 1.21	23.02 ± 1.02	22.58 ± 0.95	21.69 ± 0.78	23.54 ± 1.54
Total	49.73 ± 1.98	45.12 ± 1.80	49.39 ± 2.44	49.25 ± 2.12	48.96 ± 1.97	46.94 ± 1.70	50.91 ± 3.17
MUFA							
18:1*n*9	20.91 ± 1.27	17.65 ± 1.20	19.74 ± 1.70	21.45 ± 1.19	23.22 ± 1.39	19.98 ± 1.35	22.59 ± 2.29
18:1*n*7	4.55 ± 0.30	3.13 ± 0.19	3.69 ± 0.35	3.47 ± 0.39	4.54 ± 0.41	3.46 ± 0.30	3.91 ± 0.47
20:1*n*9	2.66 ± 0.52	1.86 ± 0.44	2.08 ± 0.41	2.12 ± 0.26	2.94 ± 0.46	2.22 ± 0.47	2.48 ± 0.43
24:1*n*9	0.09 ± 0.02	0.08 ± 0.02	0.08 ± 0.01	0.09 ± 0.03	0.13 ± 0.04	0.09 ± 0.01	0.12 ± 0.02
Total	28.21 ± 1.98	22.73 ± 1.79	25.57 ± 2.39	27.12 ± 1.43	30.84 ± 2.13	25.75 ± 2.00	29.10 ± 3.11
*n*-6 PUFA							
18:2*n*6	0.79 ± 0.03 ^a^	0.81 ± 0.03 ^ab^	0.95 ± 0.07 ^ab^	0.83 ± 0.04 ^ab^	0.91 ± 0.04 ^ab^	0.45 ± 0.02 ^b^	0.65 ± 0.05 ^a^*
20:4*n*6	14.11 ± 0.84 ^a^	10.31 ± 0.57 ^b^	10.50 ± 0.61 ^b^	10.08 ± 0.56 ^b^	9.25 ± 0.63 ^b^	8.68 ± 0.36 ^b^	8.87 ± 0.67 ^b^
22:4*n*6	3.10 ± 0.16 ^a^	2.00 ± 0.13 ^b^	1.87 ± 0.15 ^b^	1.97 ± 0.17 ^b^	1.88 ± 0.11 ^b^	1.73 ± 0.06 ^b^	1.78 ± 0.13 ^b^
Total	18.00 ± 0.98 ^a^	13.12 ± 0.65 ^b^	13.32 ± 0.73 ^b^	12.88 ± 0.71 ^b^	12.03 ± 0.72 ^b^	10.86 ± 0.34 ^b^	11.30 ± 0.81 ^b^
*n*-3 PUFA							
20:5*n*3	0.03 ± 0.00 ^a^	0.24 ± 0.01 ^b^	0.27 ± 0.03 ^b^	0.41 ± 0.02 ^b^	0.44 ± 0.03 ^b^	0.70 ± 0.02 ^b^	0.71 ± 0.06 ^b^
22:5*n*3	0.12 ± 0.01 ^a^	0.34 ± 0.02 ^b^	0.35 ± 0.03 ^b^	0.46 ± 0.02 ^b^	0.49 ± 0.03 ^b^	0.67 ± 0.05 ^b^	0.64 ± 0.06 ^b^
22:6*n*3	16.41 ± 0.70	15.83 ± 0.64	17.89 ± 1.20	18.48 ± 1.00	18.79 ± 0.96	17.05 ± 0.61	19.66 ± 1.57
Total	16.55 ± 0.71 ^a^	16.41 ± 0.65 ^ab^	18.51 ± 1.23 ^ab^	19.35 ± 1.01 ^ab^	19.71 ± 0.99 ^ab^	18.42 ± 0.60 ^ab^	20.98 ± 1.67 ^b^
*n*-6:*n*-3 ratio	1.09 ± 0.03 ^a^	0.80 ± 0.03 ^b^	0.73 ± 0.03 ^b^	0.67 ± 0.03 ^b^	0.62 ± 0.03 ^b^	0.59 ± 0.01 ^b^	0.55 ± 0.03 ^b^

Data are mean ± standard error, *n* = 12. Means without common letters within the same row differ significantly. Fatty acid concentrations in the brain were not altered by the form of DHA fed to mice except for 18:2*n*6, which was significantly higher (denoted with an *) in mice fed the 4% TG-DHA diet than the 4% PL-DHA diet. Significance is *p* < 0.05. Abbreviations: DHA, docosahexaenoic acid; MUFA, monounsaturated fatty acid; PUFA, polyunsaturated fatty acid; SFA, saturated fatty acid; *n*-3, omega-3; *n*-6, omega-6.

## References

[1] Gómez-Pinilla F. (2008). Brain foods: The effects of nutrients on brain function. Nat. Rev. Neurosci..

[2] Weiser M.J., Butt C.M., Mohajeri M.H. (2016). Docosahexaenoic Acid and Cognition throughout the Lifespan. Nutrients.

[3] Lu D.-Y., Tsao Y.-Y., Leung Y.-M., Su K.-P. (2010). Docosahexaenoic Acid Suppresses Neuroinflammatory Responses and Induces Heme Oxygenase-1 Expression in BV-2 Microglia: Implications of Antidepressant Effects for Omega-3 Fatty Acids. Neuropsychopharmacology.

[4] Orr S.K., Bazinet R.P. (2008). The emerging role of docosahexaenoic acid in neuroinflammation. Curr. Opin. Investig. Drugs.

[5] Müller C.P., Reichel M., Mühle C., Rhein C., Gulbins E., Kornhuber J. (2015). Brain membrane lipids in major depression and anxiety disorders. Biochim. Biophys. Acta.

[6] Schneider M., Levant B., Reichel M., Gulbins E., Kornhuber J., Müller C.P. (2017). Lipids in psychiatric disorders and preventive medicine. Neurosci. Biobehav. Rev..

[7] Crawford M.A., Bazinet R.P., Sinclair A.J. (2009). Fat intake and CNS functioning: Ageing and disease. Ann. Nutr. Metab..

[8] Cole G.M., Ma Q.-L., Frautschy S.A. (2010). Dietary fatty acids and the aging brain. Nutr. Rev..

[9] Singh R.B., Gupta S., Dherange P., De Meester F., Wilczynska A., Alam S.E., Pella D., Wilson D.W. (2012). Metabolic syndrome: A brain disease. Can. J. Physiol. Pharmacol..

[10] Sinclair A.J., Begg D., Mathai M., Weisinger R.S. (2007). Omega 3 fatty acids and the brain: Review of studies in depression. Asia Pac. J. Clin. Nutr..

[11] Delion S., Chalon S., Guilloteau D., Lejeune B., Besnard J.C., Durand G. (1997). Age-related changes in phospholipid fatty acid composition and monoaminergic neurotransmission in the hippocampus of rats fed a balanced or an *n*-3 polyunsaturated fatty acid-deficient diet. J. Lipid Res..

[12] Favrelère S., Stadelmann-Ingrand S., Huguet F., De Javel D., Piriou A., Tallineau C., Durand G. (2000). Age-related changes in ethanolamine glycerophospholipid fatty acid levels in rat frontal cortex and hippocampus. Neurobiol. Aging.

[13] Söderberg M., Edlund C., Kristensson K., Dallner G. (1991). Fatty acid composition of brain phospholipids in aging and in Alzheimer’s disease. Lipids.

[14] Prasad M.R., Lovell M.A., Yatin M., Dhillon H., Markesbery W.R. (1998). Regional membrane phospholipid alterations in Alzheimer’s disease. Neurochem. Res..

[15] Messamore E., Almeida D.M., Jandacek R.J., McNamara R.K. (2017). Polyunsaturated fatty acids and recurrent mood disorders: Phenomenology, mechanisms, and clinical application. Prog. Lipid Res..

[16] Ross B.M., Seguin J., Sieswerda L.E. (2007). Omega-3 fatty acids as treatments for mental illness: Which disorder and which fatty acid?. Lipids Health Dis..

[17] Maclean C.H., Issa A.M., Newberry S.J., Mojica W.A., Morton S.C., Garland R.H., Hilton L.G., Traina S.B., Shekelle P.G. (2005). Effects of omega-3 fatty acids on cognitive function with aging, dementia, and neurological diseases. Evid. Rep. Technol. Assess. (Summary).

[18] Dauncey M.J. (2013). Genomic and Epigenomic Insights into Nutrition and Brain Disorders. Nutrients.

[19] Gomez-Pinilla F., Tyagi E. (2013). Diet and cognition: Interplay between cell metabolism and neuronal plasticity. Curr. Opin. Clin. Nutr. Metab. Care.

[20] Loef M., Walach H. (2013). The omega-6/omega-3 ratio and dementia or cognitive decline: A systematic review on human studies and biological evidence. J. Nutr. Gerontol. Geriatr..

[21] Simopoulos A.P. (2006). Evolutionary aspects of diet, the omega-6/omega-3 ratio and genetic variation: Nutritional implications for chronic diseases. Biomed. Pharmacother..

[22] Janssen C.I.F., Kiliaan A.J. (2014). Long-chain polyunsaturated fatty acids (LCPUFA) from genesis to senescence: The influence of LCPUFA on neural development, aging, and neurodegeneration. Prog. Lipid Res..

[23] Cole G.M., Frautschy S.A. (2010). DHA May Prevent Age-Related Dementia. J. Nutr..

[24] Bazan N.G., Molina M.F., Gordon W.C. (2011). Docosahexaenoic acid signalolipidomics in nutrition: Significance in aging, neuroinflammation, macular degeneration, Alzheimer’s, and other neurodegenerative diseases. Annu. Rev. Nutr..

[25] Yurko-Mauro K., Alexander D.D., Van Elswyk M.E. (2015). Docosahexaenoic acid and adult memory: A systematic review and meta-analysis. PLoS ONE.

[26] Grosso G., Pajak A., Marventano S., Castellano S., Galvano F., Bucolo C., Drago F., Caraci F. (2014). Role of omega-3 fatty acids in the treatment of depressive disorders: A comprehensive meta-analysis of randomized clinical trials. PLoS ONE.

[27] Denis I., Potier B., Vancassel S., Heberden C., Lavialle M. (2013). Omega-3 fatty acids and brain resistance to ageing and stress: Body of evidence and possible mechanisms. Ageing Res. Rev..

[28] Cederholm T., Palmblad J. (2010). Are omega-3 fatty acids options for prevention and treatment of cognitive decline and dementia?. Curr. Opin. Clin. Nutr. Metab. Care.

[29] Quinn J.F., Raman R., Thomas R.G., Yurko-Mauro K., Nelson E.B., Van Dyck C., Galvin J.E., Emond J., Jack C.R., Weiner M. (2010). Docosahexaenoic acid supplementation and cognitive decline in Alzheimer disease: A randomized trial. JAMA.

[30] Andrieu S., Guyonnet S., Coley N., Cantet C., Bonnefoy M., Bordes S., Bories L., Cufi M.-N., Dantoine T., Dartigues J.-F. (2017). MAPT Study Group Effect of long-term omega 3 polyunsaturated fatty acid supplementation with or without multidomain intervention on cognitive function in elderly adults with memory complaints (MAPT): A randomised, placebo-controlled trial. Lancet Neurol..

[31] Graf B.A., Duchateau G.S., Patterson A.B., Mitchell E.S., van Bruggen P., Koek J.H., Melville S., Verkade H.J. (2010). Age dependent incorporation of 14C-DHA into rat brain and body tissues after dosing various 14C-DHA-esters. Prostaglandins Leukot. Essent. Fatty Acids.

[32] Valenzuela A., Nieto S., Sanhueza J., Nuñez M.J., Ferrer C. (2005). Tissue accretion and milk content of docosahexaenoic acid in female rats after supplementation with different docosahexaenoic acid sources. Ann. Nutr. Metab..

[33] Nguyen L.N., Ma D., Shui G., Wong P., Cazenave-Gassiot A., Zhang X., Wenk M.R., Goh E.L.K., Silver D.L. (2014). Mfsd2a is a transporter for the essential omega-3 fatty acid docosahexaenoic acid. Nature.

[34] Arabo A., Potier C., Ollivier G., Lorivel T., Roy V. (2014). Temporal analysis of free exploration of an elevated plus-maze in mice. J. Exp. Psychol. Anim. Learn. Cogn..

[35] Smedes F. (1999). Determination of total lipid using non-chlorinated solvents. Analyst.

[36] Fedor D.M., Adkins Y., Newman J.W., Mackey B.E., Kelley D.S. (2013). The effect of docosahexaenoic acid on t10, c12-conjugated linoleic acid-induced changes in fatty acid composition of mouse liver, adipose, and muscle. Metab. Syndr. Relat. Disord..

[37] Bailey S.A., Zidell R.H., Perry R.W. (2004). Relationships between organ weight and body/brain weight in the rat: What is the best analytical endpoint?. Toxicol. Pathol..

[38] Michael B., Yano B., Sellers R.S., Perry R., Morton D., Roome N., Johnson J.K., Schafer K., Pitsch S. (2007). Evaluation of organ weights for rodent and non-rodent toxicity studies: A review of regulatory guidelines and a survey of current practices. Toxicol. Pathol..

[39] Després J.-P., Lemieux I. (2006). Abdominal obesity and metabolic syndrome. Nature.

[40] Tarantino G., Colicchio P., Conca P., Finelli C., Di Minno M.N.D., Tarantino M., Capone D., Pasanisi F. (2009). Young adult obese subjects with and without insulin resistance: What is the role of chronic inflammation and how to weigh it non-invasively?. J. Inflamm. Lond. Engl..

[41] Tarantino G., Conca P., Pasanisi F., Ariello M., Mastrolia M., Arena A., Tarantino M., Scopacasa F., Vecchione R. (2009). Could inflammatory markers help diagnose nonalcoholic steatohepatitis?. Eur. J. Gastroenterol. Hepatol..

[42] Takeuchi T., Iwanaga M., Harada E. (2003). Possible regulatory mechanism of DHA-induced anti-stress reaction in rats. Brain Res..

[43] Ferraz A.C., Delattre A.M., Almendra R.G., Sonagli M., Borges C., Araujo P., Andersen M.L., Tufik S., Lima M.M.S. (2011). Chronic ω-3 fatty acids supplementation promotes beneficial effects on anxiety, cognitive and depressive-like behaviors in rats subjected to a restraint stress protocol. Behav. Brain Res..

[44] Fedorova I., Salem N. (2006). Omega-3 fatty acids and rodent behavior. Prostaglandins Leukot. Essent. Fatty Acids.

[45] Trofimiuk E., Braszko J.J. (2013). Concomitant docosahexaenoic acid administration ameliorates stress-induced cognitive impairment in rats. Physiol. Behav..

[46] Pérez M.Á., Terreros G., Dagnino-Subiabre A. (2013). Long-term ω-3 fatty acid supplementation induces anti-stress effects and improves learning in rats. Behav. Brain Funct. BBF.

[47] Su H.-M. (2010). Mechanisms of *n*-3 fatty acid-mediated development and maintenance of learning memory performance. J. Nutr. Biochem..

[48] Schipper P., Kiliaan A.J., Homberg J.R. (2011). A mixed polyunsaturated fatty acid diet normalizes hippocampal neurogenesis and reduces anxiety in serotonin transporter knockout rats. Behav. Pharmacol..

[49] Venna V.R., Deplanque D., Allet C., Belarbi K., Hamdane M., Bordet R. (2009). PUFA induce antidepressant-like effects in parallel to structural and molecular changes in the hippocampus. Psychoneuroendocrinology.

[50] Pusceddu M.M., Nolan Y.M., Green H.F., Robertson R.C., Stanton C., Kelly P., Cryan J.F., Dinan T.G. (2015). The Omega-3 Polyunsaturated Fatty Acid Docosahexaenoic Acid (DHA) Reverses Corticosterone-Induced Changes in Cortical Neurons. Int. J. Neuropsychopharmacol..

[51] Brinks V., van der Mark M., de Kloet R., Oitzl M. (2007). Emotion and Cognition in High and Low Stress Sensitive Mouse Strains: A Combined Neuroendocrine and Behavioral Study in BALB/c and C57BL/6J Mice. Front. Behav. Neurosci..

[52] Messamore E., McNamara R.K. (2016). Detection and treatment of omega-3 fatty acid deficiency in psychiatric practice: Rationale and implementation. Lipids Health Dis..

[53] Luchtman D.W., Song C. (2013). Cognitive enhancement by omega-3 fatty acids from child-hood to old age: Findings from animal and clinical studies. Neuropharmacology.

[54] Carrié I., Smirnova M., Clément M., De J.D., Francès H., Bourre J.M. (2002). Docosahexaenoic acid-rich phospholipid supplementation: Effect on behavior, learning ability, and retinal function in control and *n*-3 polyunsaturated fatty acid deficient old mice. Nutr. Neurosci..

[55] Carrié I., Clément M., de Javel D., Francès H., Bourre J.M. (2000). Specific phospholipid fatty acid composition of brain regions in mice. Effects of *n*-3 polyunsaturated fatty acid deficiency and phospholipid supplementation. J. Lipid Res..

